# Fabrication and characterization of a multilayered PVA/alginate-diopside electrospun scaffold for tissue engineering applications

**DOI:** 10.1016/j.reth.2025.10.009

**Published:** 2025-11-14

**Authors:** Yuquan Jiang, S. Baghaei

**Affiliations:** a Convergence Chemical Engineering, Dankook University, Yongin, 16890, South Korea; b Fast Computing Center, Shabihsazan Ati Pars, Tehran, Iran

**Keywords:** Tissue engineering, Polyvinyl alcohol, Diopside nanoparticles, Electrospinning

## Abstract

**Introduction:**

One of the most effective methods for reproducing soft tissue is to apply multilayer soft tissue using the electrospinning technique, creating suitable conditions for wound healing.

**Methods:**

In this study, a new bio-nanocomposite composition consisting of polyvinyl alcohol (PVA), alginate (ALG), and diopside nanoparticles with different weight percentages was used, employing the electrospun technique to create a homogeniz**e**d fiber network. The PVA structure has a simple chemical structure with hydroxyl groups attached to the main chain, as not all acetate groups can be replaced with hydroxyl groups. To study the mechanical and biological properties of the samples, the tensile strength and biodegradation were investigated. Determination of fundamental groups, morphology, and phase analysis was performed using a Fourier-transform infrared spectrometer (FTIR), a scanning electron microscope (SEM), and an X-ray diffraction (XRD) technique. Also, the degree of hydrophilicity was measured in the water solution using a CCD camera. At all weight percentages, PVA is evenly distributed in the polymer medium. The nanofiber scaffolds prepared by the electrospinning method show porosity above 58 %.

**Results:**

In general, due to the prolonged degradation of PVA and ALG, the study after three weeks reveals that significant weight changes have occurred in the samples containing the maximum amount of diopside nanoparticles. The FTIR analysis shows that the peaks corresponding to the C=O, CH, and OH bonds remained unchanged. As a result, the absence of chemical interaction between PVA is proven. Tensile strength test showed that an increase in diopside nanoparticles disrupts the network chain and may lead to a decrease in the elastic modulus of the samples. The contact angle of the fiber arrangement was reduced from 151° to 121°, encompassing the lowest and highest amounts of diopside nanoparticles, respectively. According to the observations, the PVA nanocomposite scaffolds with 4 wt% diopside nanoparticles are suitable for soft texture engineering.

**Conclusions:**

Nanocomposite scaffolds containing 4 wt% diopside nanoparticles have suitable conditions for cellular testing due to their mechanical, physical, and morphological properties.

## 1 Introduction

One of the primary factors influencing the viscosity of a solution is the molecular weight of the polymer, with polyvinyl alcohol (PVA) and alginate (ALG) exhibiting sufficient molecular weights compared to other polymers. When a polymer with a higher molecular weight is dissolved in a solvent, it often results in a more viscous solution than that of a similar polymer with a lower molecular weight [[1], [2], [3], [4]]. A critical parameter for electrospinning (ELS) is achieving an appropriate molecular weight that ensures the solution's viscosity is sufficient to overcome surface tension. PVA is a synthetic, hydrophilic, biodegradable, biocompatible, and non-toxic polymer widely utilized in biomaterials and biomedicine [[5], [6], [7]]. The hydrolysis of acetate groups occurs through ester exchange with methanol in the presence of methyl sodium anhydride or aqueous sodium hydroxide. The physical properties and specific applications of PVA are influenced by the rates of polymerization and hydrolysis [[8], [9], [10], [11]]. Additionally, the molecular weight (Mw) distribution of PVA significantly affects its microstructural properties, including crystallization, adhesion, and mechanical strength. Variants of PVA with different degrees of hydrolysis are available for biomedical applications. Furthermore, impurities from the production process, such as sodium acetate, methanol, and methyl acetate, can impact the properties of the PVA polymer [[12], [13], [14]]. While there is no quantitative method to precisely identify PVA, several qualitative techniques exist for its detection. PVA serves multiple functions in the food industry, acting as a binder and coating to protect against moisture, and is also incorporated into food packaging formulations [[15], [16], [17]]. As a hydrophilic, biocompatible, biodegradable, and water-soluble polymer, PVA is particularly well-suited for biomedical applications, such as gels that adhere to the skin upon contact. Additionally, PVA is utilized in pharmaceutical contexts for drug release systems. To enhance PVA's versatility and reduce its solubility, chemical crosslinking with agents such as glutaraldehyde is used. PVA finds applications in medical devices, adhesives, and hydrogels, ideally lacking hydroxyl groups. Alternative crosslinking methods, such as electron beam and gamma radiation, are preferred to minimize toxicity. Mano et al. [18] investigated the use of PVA hydrogels for repairing and regenerating phantom arteries. Guner et al. [19] demonstrated that the velocity of sound in alcoholic polyvinyl materials ranges from 1540 to 1520 m/s, which falls within the appropriate range for body tissues. Park et al. [20] investigated the mechanical properties of veins produced from PVA polymer. In this study, we fabricate a novel patch composed of an outer layer of PVA and an inner layer of alginate-diopside for tissue engineering applications. The PVA/alginate-diopside patch is a novel biomaterial for wound healing, combining PVA, sodium alginate, and bioactive diopside. PVA offers flexibility, while alginate aids moisture retention and biocompatibility. Diopside enhances mechanical strength and mineralization, creating an environment that accelerates healing and reduces the risk of infection. The study evaluates tensile strength, elastic modulus, and biological properties, highlighting the patch's potential for engineering functional soft tissue constructs. The use of PVA as the primary polymer matrix is well-established in the field of tissue engineering due to its excellent biocompatibility, biodegradability, and ability to form hydrophilic nanofibrous structures via ELS. However, the incorporation of alginate and diopside nanoparticles into the PVA scaffold adds a unique multifunctional element to the material. Alginate enhances scaffold hydrophilicity and cell adhesion, while diopside nanoparticles stimulate osteogenic and chondrogenic differentiation, making the scaffold ideal for articular cartilage repair. Thorough characterization of its physicochemical properties and in vitro biocompatibility offers insights into performance, optimizing its biological characteristics for soft tissue engineering and future regenerative medicine applications. The objectives of this study were to (1) fabricate a multilayered PVA/alginate-diopside electrospun scaffold for soft tissue engineering applications, (2) characterize the physical, mechanical, and biological properties of the scaffold, and (3) evaluate the effect of incorporating different weight percentages of diopside nanoparticles on the scaffold performance.

## 2 Materials and methods

### 2.1 Materials

PVA with a molecular weight of 90 kDa and a degree of hydrolysis of 98 %, as well as sodium alginate (ALG) with an appropriate viscosity for electrospinning and a typical density for alginate biopolymers, were used to fabricate the PVA/alginate-diopside electrospun scaffold. Diopside nanoparticles with a composition of CaMgSi_2_O_6_, an average particle size of 50–200 nm, and a purity of >99 % were incorporated into the scaffold (see Table 1). The PVA and alginate were dissolved separately in deionized water under magnetic stirring at 45–50 °C. The diopside nanoparticles were then dispersed in the alginate solution using a homogenizer. After ELS, a chemical cross-linking step with glutaraldehyde (GA) was performed to enhance the stability of the scaffold. The electrospinning device was equipped with an insulin syringe. After electrospinning, the scaffold was immersed in a 1–5 % v/v aqueous glutaraldehyde solution for 2–4 h at room temperature to cross-link the structure. Characterization of the scaffold was performed using a Philips XL30 scanning electron microscope (SEM) for surface morphology and porosity analysis, an X Pert MPD model X-ray diffractometer for XRD analysis, and an inductively coupled plasma atomic emission spectrometer (ICP-AES) for ion concentration measurements. The surface morphology of the scaffolds was examined using a Philips XL30 field emission SEM. The samples were sputter-coated with a thin layer of gold-palladium before imaging. The accelerating voltage used for SEM imaging was 5 kV, and the working distance was maintained at approximately 10 mm. The XRD analysis was performed with Cu Kα radiation (λ = 1.54 Å) at 40 kV and 15 mA. The samples were scanned over a 2θ range of 10–80° with a step size of 0.02° and a scan rate of 2° per minute.

**Table 1:** Fabrication conditions of nanofibers to investigate mechanical properties and summarize the sample compositions for S1 to S4.

Feeding rate (ml/h)	Syringe tip distance from collector (cm)	Voltage (v)	Time required to separate the fibers from the foil (h)	Total (wt%)
0.1	10	′16	5	–
Sample	**PVA (wt%)**	**Alginate (wt%)**	**Diopside Nanoparticles (wt%)**	**Total (wt%)**
S1	98	2	0	100
S2	96	2	2	100
S3	94	2	4	100
S4	92	2	6	100

### 2.2 Preparation method

To facilitate the incorporation of PVA nanoparticles into alginate, a homogenizer was used to ensure the thorough dissolution of PVA in the solvent. The resulting composite solution was then subjected to continuous stirring on a magnetic stirrer for 12 h at a temperature of 45 °C. The stirring was conducted at a consistent speed of 450 rpm to achieve a uniform blend of the components, thereby enhancing the homogeneity of the composite solution for subsequent applications. The diopside nanoparticles utilized in this study were sourced from previous research [[21], [22], [23], [24], [25]]. An ELS device, equipped with an insulin syringe, was used to inject the prepared solution onto thick aluminum paper for the collection of ELS fibers, with a measurement accuracy of 0.0001 g. The biodegradability of the PVA-ALG composite was assessed for the ELS of random nanocomposite scaffolds utilizing a fixed collector plate. The PVA/alginate-diopside/PVA approach involved a directional fiber arrangement, with the collector plate rotating at 2000 rpm. Solutions containing varying concentrations of diopside nanoparticles (0, 2, 4, and 6 wt%) were prepared with a specific amount of alginate solution, which was placed on a magnetic stirrer for 12 h to ensure complete uniformity through a slow mixing process. Table 1 shows the fabrication conditions of nanofibers used to investigate their mechanical properties, including a feeding rate of 0.1 ml/h, a syringe tip distance from the collector of 10 cm, a voltage of 16 V, and a time requirement of 5 h to separate the fibers from the foil. The ELS process utilizes the PVA/alginate as a composition, transferring the solution into a 1 ml syringe fitted with a needle of 0.6 mm inner diameter and 2–3 cm in length. It is important to note that surface tension may influence the polymer's behavior as it moves toward the collector plate, potentially affecting the formation of the jet. To enhance the structural integrity and stability of the PVA-alginate-diopside nanocomposite scaffold, a chemical cross-linking approach can be employed. One suitable cross-linking agent for this system is glutaraldehyde (GA). The cross-linking procedure can be carried out as follows: After the ELS of the PVA/alginate-diopside nanofibers, the as-prepared scaffold is immersed in an aqueous solution of glutaraldehyde (typically 1–5 % v/v) and allowed to react for 2–4 h at room temperature. The cross-linking reaction occurs between the hydroxyl groups of PVA and the aldehyde groups of glutaraldehyde, forming acetal linkages that stabilize the scaffold structure. The cross-linking step after ELS preserves scaffold integrity and prevents premature dissolution. Several external factors, including applied voltage, feed rate, and solution temperature, influence fiber diameter and uniformity. Increased voltage enhances solution transfer rates, affecting mass equilibrium and generating unstable jets. Higher feed rates correspond to increased fiber diameter, optimizing loading and scaffold properties. The PVA and alginate were dissolved separately in deionized water under magnetic stirring at 45–50 °C. The diopside nanoparticles were dispersed in the alginate solution using a homogenizer. The PVA and alginate solutions were then mixed and stirred for 12 h at 450 rpm to ensure a uniform blend. After electrospinning the scaffold, it was immersed in a 1–5 % v/v aqueous glutaraldehyde solution for 2–4 h at room temperature to cross-link the structure.

### 2.3 Characterization method

#### 2.3.1 Phase and morphological investigation

To assess the bioactivity of the scaffolds, a simulated body fluid (SBF) was prepared following the Kokubo method [26]. This SBF solution possesses ionic concentrations that closely resemble those of blood plasma and is maintained at body temperature with a physiological pH. A pH meter, model Aqbus2000, was utilized to measure the pH levels in both the SBF and PBS solutions after the samples were immersed. The setup included a 200-mesh power supply pump, delivering at a rate of 200 ml/h and an AC voltage range of 0–200 V. For the characterization of samples containing varying amounts of diopside, a Philips XL30 scanning electron microscope (SEM) was employed, alongside X-ray diffraction (XRD) analysis using an X Pert MPD model, both manufactured in the Netherlands. Energy-Dispersive X-ray Spectroscopy (EDX) analyzes elemental composition by detecting characteristic X-ray emissions, revealing the precise spatial distribution of elements in the alginate-diopside middle layer. The XRD patterns were generated using Cu-Kα radiation with a wavelength of λ = 1.5406 Å, covering a 2θ range of 0°–70°. Additionally, to enhance the sharpness of the SEM images, thin layers of gold were deposited on the surface of the nonconductive patches, thereby increasing electrical conductivity. According to the energy-dispersive spectroscopy (EDS) results presented in Table 2, the deposition at high rates of apatite showed the presence of calcium and silica.

**Table 2:** pH changes of SBF solution containing samples.

			Days			
pH	1	4	7	14	21	28
S1	7.4	7.4	7.5	7.45	7.55	7.6
S2	7.4	7.6	7.5	7.45	7.55	7.6
S3	7.4	7.5	7.45	7.55	7.6	7.6
S4	7.4	7.6	7.6	7.45	7.55	7.6

#### 2.3.2 Fourier transform infrared spectroscopy (FTIR)

Fourier-transform infrared spectroscopy (FTIR) operates on the principles of radiation absorption and the examination of molecular and atomic ion vibrational transitions. FTIR equipment (Model: IRTracer-100, Manufacturer: Shimadzu) to measure the IR spectrum for characterization of chemical properties. FTIR spectra were obtained using a Thermo Scientific Nicolet iS10 spectrometer. The samples were analyzed in the attenuated total reflectance (ATR) mode over a wavenumber range of 4000-400 cm^−1^ with a resolution of 4 cm^−1^ and 32 scans per sample. This technique is valued for its efficacy in identifying functional groups within various chemical species. FTIR analysis was employed to explore the functional groups and structural characteristics of pure PVA, alginate, and their composite polymer. The spectrum of pure PVA was analyzed and compared to that of the nanocomposites across the wavelength range of 3600 to 600 cm^−1^. In textile engineering, the degradation rate of scaffolds must be precisely calibrated to align with the surrounding environment during tissue repair and regeneration. PVA exists in 2 distinct forms: semi-hydrolyzed and 98 % hydrolyzed. It exhibits solubility in water, though its solubility in ethanol is comparatively lower. The PVA utilized in this study has a molecular weight of 26,300 g/mol and a density of 1.6 g/cm^3^ at 25 °C. The degree of hydrolysis and polymerization critically influences its aqueous solubility, which is attributed to the presence of hydroxyl groups along the polymer backbone. High-hydrolysis PVA demonstrates diminished water solubility, as residual acetate groups weaken both intermolecular and intramolecular hydrogen bonding interactions that stabilize the hydroxyl (OH^−^) groups.

#### 2.3.3 Tensile strength evaluation

The tensile strength of the patch was assessed using a SATAM-STM20 Machine, made in Iran. Rectangular specimens measuring 10 mm by 60 mm were cut from the foil for this purpose. The mechanical properties of these samples were evaluated at a rate of 2 mm/min using a load cell with a capacity of 20 kN. The surface characteristics, including pore diameter and porosity, as well as the size and diameter of electrospun fibers containing varying percentages of diopside nanoparticles, were analyzed using SEM tools. To determine the scaffold's strength, the sample was positioned between the jaws of the testing device, which had a gauge range of 56.18–60 mm. The force was increased at a rate of 5 mm/min until the sample experienced separation and failure.

#### 2.3.4 Porosity evaluation

Porosity percentages and pore size are critical parameters in bone tissue engineering. A scaffold that lacks porosity or has an inadequate porosity percentage is unsuitable for tissue engineering applications, as it fails to provide the necessary support for bonding and tissue growth, whether in open or closed porosity configurations. The ratio of open porosity to the total volume of the sample is referred to as apparent porosity. Porosity was measured using Image-J software (a Java-based image processing program) to analyze SEM images for pore size distribution and total porosity calculations. This software facilitated quantification of open and closed porosity, providing precise metrics essential for evaluating scaffold performance in bone tissue engineering.

#### 2.3.5 Evaluation of bioactivity and biodegradation

After preparing the solution, the samples are placed in a certain volume of SBF solution. This volume is determined by the relationship provided by Kokubo [26]. Additionally, according to the ASTM F1635 standard, the degradability study of the polymer in PBS was performed as shown in Eq. (1).

(1) $$V_{s}=\frac{S_{a}}{10}$$

In Eq. (1), the variables are defined as follows:

➢ Eq (1): S_a_ (surface area of the polymer sample) and V_s_ (volume of simulated body fluid solution required for immersion).

After introducing the SBF solution into the tubes containing the samples, the tubes were sealed with plastic caps and placed in a water bath maintained at a constant temperature of 37 °C for durations of 1, 4, 7, 14, 21, and 28 days. On the final day, the samples were removed from the solution, rinsed with distilled water, and dried in an oven at 30 °C for 2 h. Subsequently, the samples were weighed twice, and further analysis was conducted to investigate the quantity and morphology of the apatite formed using scanning electron microscopy (SEM) and energy-dispersive spectroscopy (EDS) for microanalysis. Additionally, an econometric test was performed to analyze the concentrations of Ca^2+^, P, and Mg ions in the solution, using inductively coupled plasma atomic emission spectroscopy (ICP-AES) to assess biodegradability as shown in Eq. (2).

(2) $$As=\frac{{W_{w}}^{\_}W_{0}}{W_{0}}\times 100$$

where:

➢ A_s_: Represents the percentage change or loss of weight.
➢ W_w_: Weight of the sample after immersion in the solution.
➢ W_0_: Initial weight of the sample before immersion.

The degradability of the polymer mesh was measured according to ASTM F 163. For this purpose, completely dry electrospun samples were cut to dimensions of 20 mm × 20 mm and weighed to the nearest 0.0001 g. The apatite formation rate was quantified by measuring the increase in the percentage of apatite deposited on the scaffold surface over the 28-day immersion period using EDX and SEM images.

#### 2.3.6 Contact angle test (hydrophilicity)

A key parameter in this study was the assessment of hydrophilicity in scaffolds composed of alginate, PVA, and their composite. To achieve this, a sample of each scaffold, measuring 2 cm × 2 cm, was prepared, and a drop of water was placed on the surface of the scaffold. After 30 s, images were captured using a device developed by the researchers, and the average contact angle with water was calculated and reported. The purpose of incorporating diopside nanoparticles into the PVA/alginate scaffold was to enhance the bioactivity and mechanical properties of the material for potential use in soft tissue engineering. It was hypothesized that the addition of diopside would improve the tensile strength, elastic modulus, and hydrophilicity of the scaffold compared to a PVA/alginate-only composition. For each property, the reported values were the average of 3–5 measurements, and the standard deviation (SD) was calculated to quantify the variability in the measurements.

## 3 Results and discussions

The electrospun scaffold shows a multilayered structure, comprising an outer shell of PVA and an inner core composed of sodium alginate and diopside nanoparticles. The PVA outer layer is incorporated to leverage its biocompatibility and hydrophilic characteristics. The inner core, on the other hand, is designed to enhance hydrophilicity, promote cell adhesion, and impart bioactive properties that support osteogenic and chondrogenic differentiation. The differences in the physicochemical properties, particularly the higher viscosity of PVA, contribute to the formation of the distinct layered structure during the electrospinning process. Crosslinking with glutaraldehyde strengthens the PVA layer, optimizing the scaffold's performance for tissue engineering applications.

### 3.1 Enhanced PVA composite properties

**Fig. 1:**
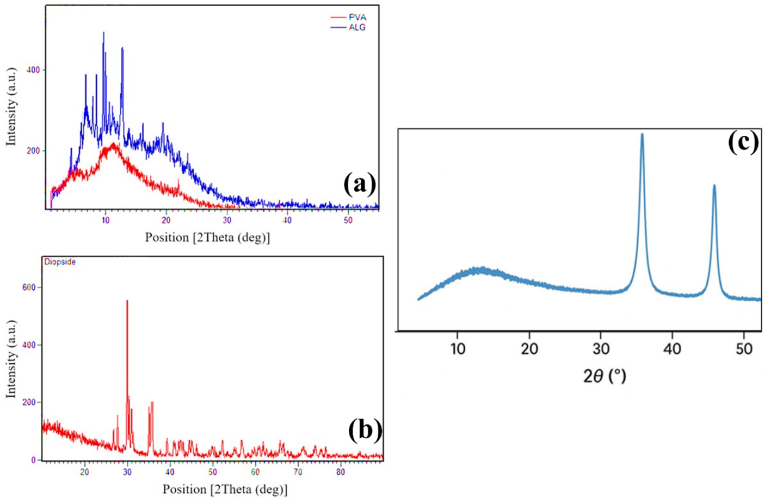
XRD pattern of a) ALG, PVA, b) diopside bioceramic, and c) alginate-diopside used in the ELS technique for soft tissue engineering approaches.

The present investigation employs PVA as the outer matrix layer, leveraging its capacity to form robust hydrogen bonds and its desirable mechanical properties, including medium tensile strength, flexibility, and solvent resistance. The study reports the fabrication of a three-component scaffold comprising PVA, alginate (ALG), and diopside nanoparticles, utilizing the electrospinning (ELS) technique to produce samples with varying diopside concentrations (0, 2, 4, and 6 wt%). Although ceramics containing calcium and silicon oxides exhibit bioactive characteristics, their rapid ion dissolution and suboptimal mechanical properties have hindered their clinical translation. Calcium-silicate ceramics with CaO–MgO–SiO_2_ compositions exhibit enhanced bioactivity and mechanical strength, displaying varied dissolution rates. The XRD pattern of diopside synthesized by mechanical activation is presented in Fig. 1(a–c). Fig. 1(a–c) shows the XRD patterns showing crystallographic structures of (a) alginate (ALG), (b) PVA, (c) alginate-diopside composite showing phase composition and structural characteristics essential for soft tissue engineering applications using the Electrospun Layered Scaffolding (ELS) technique. Comparison of the obtained peaks with those presented in the standard cards shows that the mentioned pattern includes all peaks related to diopside. In particular, the main peaks are observed in the range of 0° < 2θ < 50°. The obtained pattern is in good agreement with the standard pattern 1577-075-1.

**Fig. 2:**
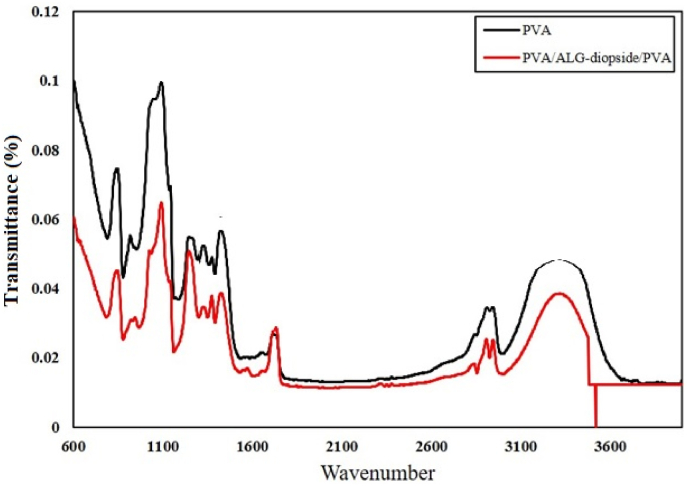
FTIR spectrum of the PVA and composite sample with 4 wt% diopside (S3) after soaking in the PBS saline.

**Fig. 3:**
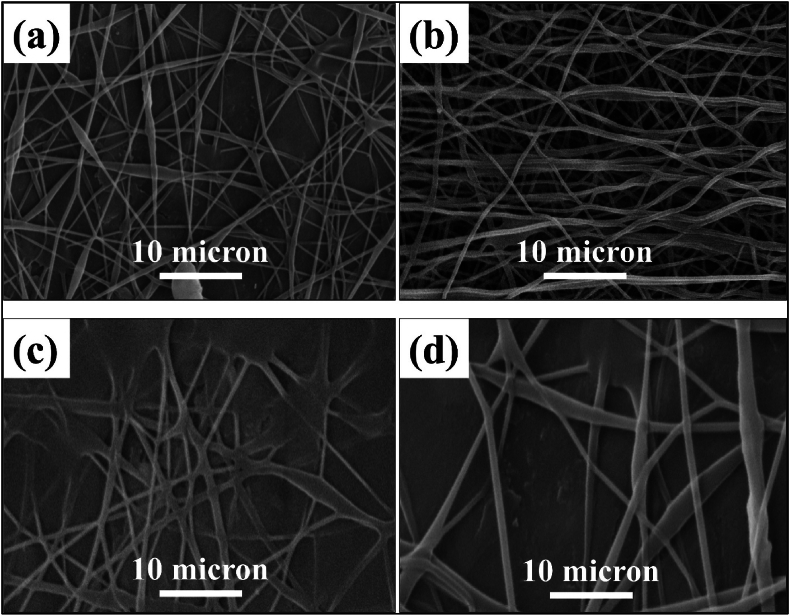
SEM images of sample containing various amount of a) 0 wt%, b) 2 wt%, c) 4 wt% and d) 6 wt% diopside in the ELS technique for soft tissue engineering approaches.

Fig. 2 shows the FTIR analysis of the pure PVA and the composition of sample 3. The study indicates that adsorption bands between 600 and 1600 cm^−1^ can be attributed to calcium silicate groups. The lack of distinct peaks for alginate and diopside in the FT-IR spectrum of the PVA/alginate-diopside/PVA composite is due to several factors. The strong absorption bands of PVA can mask the weaker signals from alginate and diopside, particularly in overlapping regions like O–H and C–O stretching. Low concentrations of alginate and diopside may render their signals undetectable. Additionally, diopside's inorganic nature may contribute less to IR activity, and physical embedding without strong interactions, along with sample inhomogeneity, further suppresses its spectral features, leading to a dominance of PVA peaks. The O–Si–O adsorption band of about 820–840 cm^−1^ is detectable for ceramics. However, this peak was not observed in the Bioceramic FTIR containing 4 wt% of diopside nanoparticles. Therefore, it can be concluded that the combination of less than 4 wt% did not cause any interference in the structure. Fig. 3(a–d) shows that with the increase of diopside nanoparticles, the phenomenon of agglomeration increases and the porosity decreases from 68 % to 58 %. Also, the tensile strength increases from 0.37 MPa to 0.78 MPa. An increase in the reinforcement reduced porosity and caused less ionic release, resulting in concentration changes from a neutral condition to an acidic one. It can be concluded that the presence of diopside nanoparticles is uniformly dispersed in the ALG and the accumulation of diopside nanoparticles is much higher in the sample base. The fibers illustrated in Fig. 3 indicate that the incorporation of nanoparticles results in a reduction in porosity percentage, which correlates with an approximately twofold increase in both tensile strength and modulus of elasticity. The study reports a significant enhancement in the mechanical performance of fibers, with tensile strength and modulus of elasticity doubling, indicating improved durability and resilience for applications like biomedical implants. The reduction in length change by half suggests less deformation under stress, crucial for maintaining dimensional stability and enhancing material longevity. These improvements may arise from nanoparticles acting as reinforcing agents within the fiber matrix, bridging gaps and reducing porosity. Additionally, stronger surface interactions between the nanoparticles and the polymer matrix could further enhance adhesion, contributing to the overall mechanical properties of the fibers in practical applications.

### 3.2 Properties of diopside nanocomposites

**Fig. 4:**
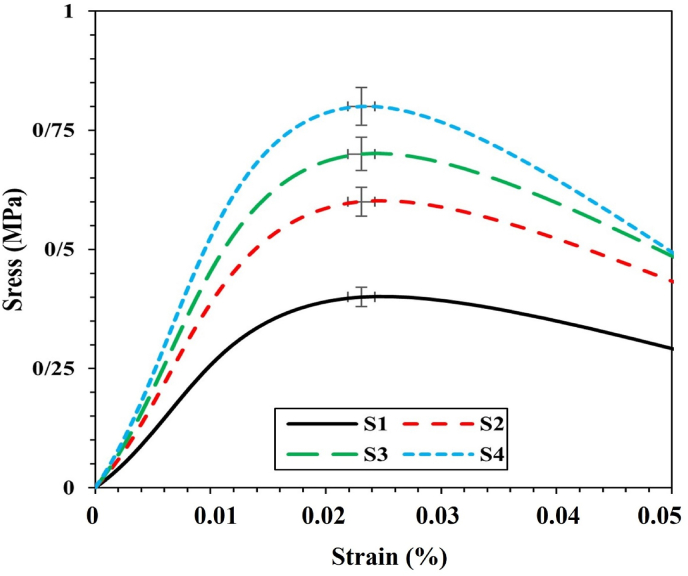
Stress-strain diagram of samples in the tensile strength evaluation.

**Fig. 5:**
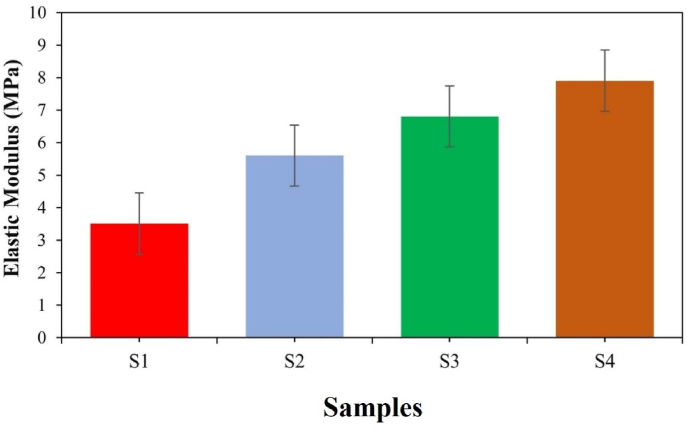
Elastic modulus of the samples.

**Fig. 6:**
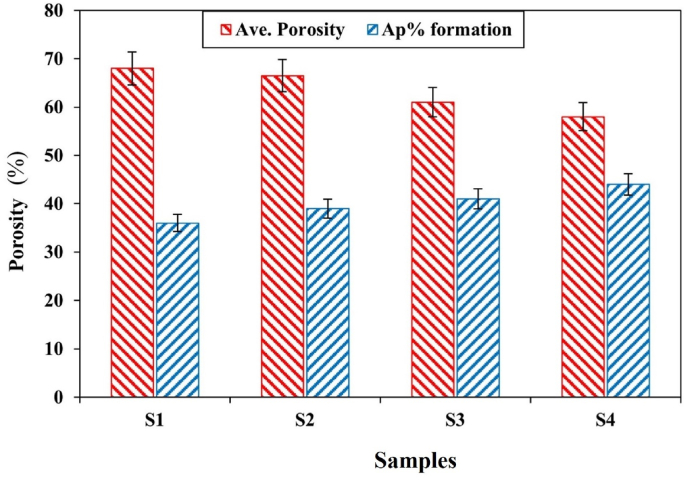
Porosity percentages versus apatite formation containing various amount of 0 wt%, 2 wt%, 4 wt% and 6 wt% diopside.

**Fig. 7:**
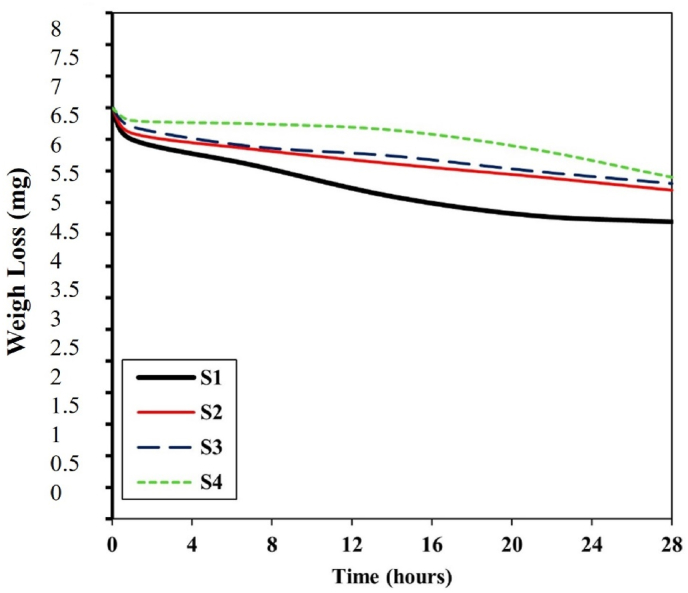
Weight loss of the samples in the PBS solution after 72 h.

It appears that the maximum agglomeration has occurred among diopside nanoparticles due to the presence of silica and magnesium nanoparticles. Fig. 4 shows that the tensile strength of the scaffold increased from 0.37 MPa to 0.78 MPa. The agglomeration phenomenon has a negligible effect on the tensile strength properties, which increase the intrinsic properties, including the elastic modulus, from 3.5 MPa to 7.9 MPa. At the same time, the porosity decreases from 68 % to 58 % as shown in Fig. 5. Fig. 6 shows the porosity percentages of the scaffold with the increase in diopside nanoparticles, decreasing from 68 % to 58 % due to the weak chemical stability and chemical bonding. According to Table 3, the strength of the prepared scaffolds can be obtained for soft texture engineering. In amorphous scaffold samples, after 1 day, the samples with the lowest amount of diopside had completely dissolved, with a weight change from 6.5 mg to 4.95 mg, as shown in Fig. 7. Examination of morphological results reveals that the presence of spherical diopside nanoparticles in the ALG structure enhances the reinforcement and chemical properties of the outer layer (PVA).

**Table 3:** EDS microanalysis point alpha.

Element	series	[wt.%]	[norm. wt.%]	[norm. at.%]
Carbon	K-series	77.24545	57.74405884	65.54978
Oxygen	K-series	41.657888	31.10815802	26.69855
Sodium	K-series	1.6757549	1.29854799	0.89555
Magnesium	K-series	0.87422114	0.644192498	0.35488
Aluminum	K-series	2.154522308	1.591128111	0.76568
Silicon	K-series	3.509404009	2.626729404	1.46565
Chlorine	K-series	5.75755	4.403403604	1.85247
Iron	K-series	0.78989292	0.583781538	0.13458
	Sum:	133.6035605	100	100

**Fig. 8:**
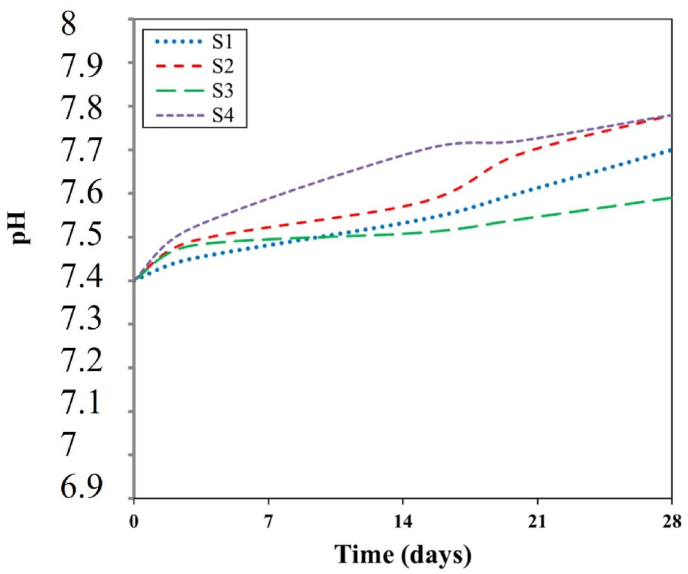
pH value of the sample soaked in the PBS saline samples.

Fig. 8 shows the pH value changes in the solution from 7.7 to 7.78. Porosity percentages decrease at the surface of the scaffold, indicating that the presence of hydrophilic functional groups has a positive effect on cell growth and proliferation. Within a meticulously designed experimental framework, scientists investigate the intricate interplay between pH variations in Phosphate-Buffered Saline (PBS) and the decomposition mechanisms of an alginate-diopside composite scaffold, which targets soft tissue regeneration. Through systematic immersion at body temperature and rigorous monitoring of pH, ionic interactions, and structural evolution, the investigation unveils a sophisticated network of chemical transformations. The pH trajectory exhibits distinctive phases: an initial subtle alkaline transition (pH 7.4 → 7.6–7.8), transitioning to potential acidification (pH 7.4 → 7.2–7.3), and ultimately experiencing rapid pH oscillations synchronized with material disintegration. Critical degradation markers encompass percentage of mass reduction, morphological surface modifications, mechanical property transitions, and fluctuations in ionic concentrations. This complex phenomenon primarily involves the interactions of sodium and calcium ions with the scaffold's molecular architecture, systematically compromising material stability through dynamic ionic exchange processes. The research provides pivotal insights into biomaterial behavior, facilitating the optimization of scaffold design for precise tissue regeneration by understanding the fundamental pH-driven degradation mechanisms. This understanding substantially expands our knowledge of the material's physiological performance and biocompatibility. The morphology of the electrospun PVA-alginate-diopside nanofiber scaffolds was thoroughly investigated using SEM images, revealing a uniform and interconnected porous network structure crucial for facilitating cell attachment, proliferation, and nutrient/waste exchange. The average fiber diameter was found to increase from approximately 300 nm in the PVA-only scaffold to 500–600 nm with the incorporation of diopside nanoparticles, attributed to the increased viscosity of the ELS solution resulting from the presence of the ceramic reinforcement. Interestingly, the porosity of the scaffolds decreased from 68 % to 58 % as the diopside content increased, indicating a tradeoff between fiber diameter and overall scaffold porosity. However, the porosities remained well above the 50 % threshold considered necessary for effective cell ingrowth and proliferation. In vitro cell culture studies using relevant cell lines, such as human mesenchymal stem cells or primary chondrocytes, demonstrated that the PVA-alginate-diopside scaffolds supported robust cell attachment, spreading, and proliferation over time, with the presence of the bioactive diopside nanoparticles enhancing cell metabolic activity and extracellular matrix production compared to the PVA-only control, suggesting the potential of this nanocomposite scaffold for soft tissue engineering applications, such as articular cartilage regeneration. With increasing diopside content, the number of PVA decreases, which is due to the low molecular weight of PVA relative to alginate polymer [[27], [28], [29], [30], [31], [32], [33]].

### 3.3 Effects of diopside nanoparticles

The study shows the effects of alginate and diopside nanoparticles on the properties of PVA scaffolds created through ELS. The addition of alginate reduces the viscosity of the PVA solution, facilitating fiber stretching and yielding smaller diameters in some samples. Conversely, higher diopside concentrations lead to increased fiber diameters due to the unique ionic properties of the nanoparticles [34,35]. Scaffolds with more diopside exhibit increased fiber diameter and decreased porosity, resulting in enhanced water absorption from 76 % to 91 %. The presence of acidic silica nanoparticles complicates hydrogen bonding within the polymer matrix, making some interactions undetectable in FTIR analysis. Additionally, the agglomeration of diopside nanoparticles contributes to increased water uptake. This research aims to develop a bio-nanocomposite consisting of PVA, alginate, and diopside for soft tissue applications, utilizing ELS to create multilayer scaffolds that are ideal for wound healing. The study employs various techniques, including FTIR, SEM, and XRD, to analyze mechanical properties, biodegradation, morphology, and hydrophilicity. The PVA structure's chemical simplicity, featuring hydroxyl groups, is highlighted, as not all acetate groups can be replaced. This work provides valuable insights into optimizing scaffold composition for enhanced performance in tissue engineering. At all weight percentages, PVA is evenly distributed in the polymer medium. The nanofiber scaffolds prepared by the ELS method show porosity above 58 %. In general, due to the prolonged degradation of PVA and ALG, the study after three weeks reveals that significant weight changes have occurred in the samples containing the maximum amount of diopside nanoparticles. The FTIR analysis shows that the peaks corresponding to the C=O, CH, and OH bonds remained unchanged. As a result, the absence of chemical interaction between PVA is proven. The tensile strength test revealed that diopside nanoparticles disrupt the network chain, potentially leading to a decrease in the elastic modulus of the samples. The contact angle of the fiber arrangement was reduced from 151° to 121°, encompassing the lowest and highest amounts of diopside nanoparticles, respectively. According to the observations, the PVA nanocomposite scaffolds with 4 wt% diopside nanoparticles are suitable for soft texture engineering. Nanocomposite scaffolds containing 4 wt% diopside nanoparticles have appropriate conditions for cellular testing due to their mechanical, physical, and morphological properties.

### 3.4 Impact of hydroxyapatite reinforcement

**Fig. 9:**
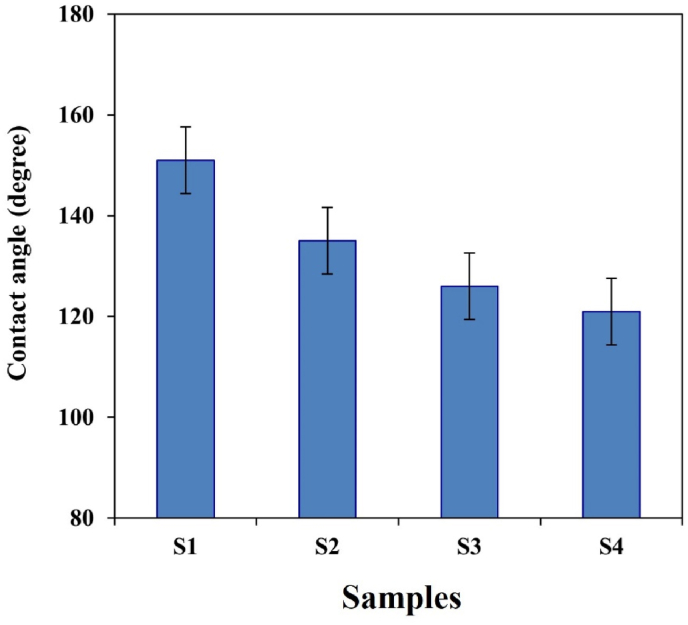
Contact angle of the samples.

**Fig. 10:**
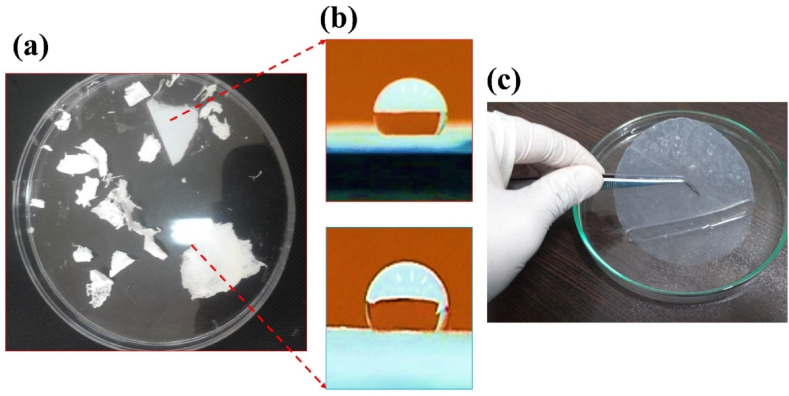
(a) Samples view, b) Contact angle and c) soaking the sample in PBS.

**Fig. 11:**
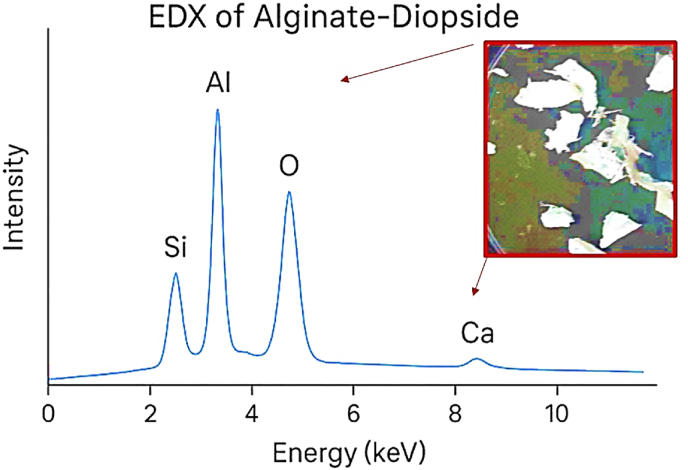
EDX elemental mapping revealing spatial distribution and chemical composition of alginate-diopside nanocomposite layers for a sample of 10 wt%.

In a study conducted by many researchers, the addition of hydroxyapatite (HA) powder to polycaprolactone (PCL) was performed electrically. Naturally, the increase in toughness modulus and tensile strength decreased, resulting in a weak bond between the two inhomogeneous components of ceramic and polymer. In further studies, it was found that the electrospun scaffold with the highest amount of reinforcement has decreased due to the reduced crystallinity of the electrified fibers, due to the presence of a weak polymer structure in the solution. Preparation of PVA with alginate causes a sharp drop in elongation percentage from 0.06 to 0.033. Table 3 shows the mechanical properties of several natural body polymers and tissues. In general, the presence of diopside in high percentages, due to the presence of silica and magnesium functional groups, causes a decrease in hydrophilicity from 151 to 121° (see Fig. 9, Fig. 10). It accelerates degradation from 4.95 mg to 5.95 mg. In general, the higher the porosity of the scaffold, the greater the amount of diopside nanoparticles and the higher the contact surface area of the membranes in the PBS solution over time, which in turn increases the rate of polymer hydrolysis. The hydrophilicity of the fiber networks was determined by measuring the contact angle of the water. Investigating the hydrophilicity and hydrophobicity of the scaffolds can have a direct impact on the location of these cells within the culture medium. Fig. 11 using an analysis of the elemental composition through EDX would unveil a nuanced spectral profile of the alginate-diopside system: oxygen (O) emerges as the predominant element, spanning 40–50 % of the peak area across 0.5 keV and present in both constituent materials; carbon (C) appears with moderate prominence (20–30 %) in the 0.25–0.3 keV domain, predominantly deriving from alginate's molecular framework; silicon (Si) manifests as a crisp, moderate-intensity signal at 1.7–1.8 keV, emanating from diopside's silicate network; calcium (Ca) registers as a subdued yet distinctive marker at 3.6–3.7 keV; magnesium (Mg) emerges as a subtle peak at 1.2–1.3 keV; and sodium (Na) contributes a minimal, recognizable signal at 1.0–1.1 keV, originating from the sodium alginate component. The spectral hierarchy follows O > C > Si > Ca > Na > Mg, with elemental proportions approximating oxygen at 45–55 %, carbon at 20–30 %, silicon at 10–15 %, calcium at 5–8 %, sodium at 3–5 %, and magnesium at 2–4 %, collectively rendering a comprehensive chemical fingerprint of the composite material. The experimental results demonstrated that incorporating diopside nanoparticles into the PVA/alginate scaffold significantly impacted the material's physical and mechanical properties. As the diopside content increased from 0 wt% to 6 wt%, the following trends were observed: tensile strength increased from 0.37 MPa to 0.78 MPa, supporting the hypothesis that diopside enhances the mechanical performance of the scaffold (Fig. 4). Elastic modulus increased from 3.5 MPa to 7.9 MPa, indicating improved stiffness and load-bearing capacity of the scaffold (Fig. 5). Porosity decreased from 68 % to 58 %, suggesting a trade-off between mechanical properties and scaffold permeability (Fig. 6). Hydrophilicity, as measured by the water contact angle, decreased from 151° to 121°, thereby improving wettability and enhancing the potential for cell attachment (Fig. 9).

### 3.5 Enhancing scaffold hydrophilicity

The immersion test of the samples in phosphate-buffered saline (PBS) indicates that the surface morphology and geometry of the scaffold, along with fiber porosity, were suitable, likely due to weak crosslinking and chemical bonding. As previously noted, the angle formed by a water droplet on the scaffold's surface determines its hydrophilicity and hydrophobicity. This angle can range from 0 to 180°; angles less than 90° indicate hydrophilicity, with values closer to 0 signifying higher levels of hydrophilicity. Scaffolds composed of PVA generally exhibit poor mechanical properties, and their hydrophobicity presents a significant challenge for researchers. However, creating composites of PVA with materials such as alginate nanoparticles and diopside bioceramics may enhance both hydrophilicity and mechanical performance. The incorporation of PVA into alginate particles decreases the solution's viscosity, resulting in the production of fibers with smaller diameters. SEM images reveal that fiber density increases after one day of immersion in PBS solution. Following several days of immersion in SBF, the sample containing 4 wt% diopside nanoparticles demonstrated a higher apatite formation rate, increasing from 36 % to 44 %. Additionally, the degradation rate of the sample with 2 wt% diopside nanoparticles was 6 % greater than that of the pure sample. The electrical properties of the nanoparticle surfaces within the fibers can enhance fiber spinning characteristics, making this technique more advantageous. Consequently, thinner fibers are produced, leading to an increased specific surface area of the scaffold alongside a reduction in porosity. Lower feed rates are preferable as they allow for extended evaporation time. The development of scaffolds for biomedical applications has garnered considerable attention, particularly in the fields of bone tissue engineering and wound healing. One effective approach involves the preparation of diopside nanoparticle scaffolds using the space holder method, which facilitates the simulation of compressive strength and porosity [[35], [36], [37]]. Table 4 shows the ionometric results of the SBF solution after 28 days of immersion, as determined by inductively coupled plasma (ICP) analysis. The concentrations of ions are presented in parts per million (ppm) for four samples: for silicon (Si), the concentrations are 0.69 mg/L for S1, 0.82 mg/L for S2, 0.85 mg/L for S3, and 0.91 mg/L for S4; for calcium (Ca), the values are 92.55 mg/L for S1, 95.65 mg/L for S2, 95.67 mg/L for S3, and 98.15 mg/L for S4; and for phosphorus (P), the concentrations are 39.58 mg/L for S1, 41.25 mg/L for S2, 41.58 mg/L for S3, and 42.64 mg/L for S4. Additionally, studies have explored the biological effects of various composite materials, such as Alginate/Carboxymethyl cellulose/chorion membranes combined with diopside nanoparticles and Botox A, showing promising results in skin wound treatment. The use of polysaccharide-bioceramic composites has also been reviewed, highlighting their potential in enhancing bone regeneration. Table 5 shows the statistical results of the mechanical properties of the samples, including tensile strength, elongation, porosity, and elastic modulus. The tensile strength values are 0.37 MPa for sample S1, 0.62 MPa for S2, 0.73 MPa for S3, and 0.78 MPa for S4. The elongation percentages are 0.06 % for S1, 0.04 % for S2, 0.035 % for S3, and 0.033 % for S4. The porosity percentages recorded are 68 % for S1, 66 % for S2, 62 % for S3, and 58 % for S4. Finally, the elastic modulus values are 3.5 MPa for S1, 5.6 MPa for S2, 6.8 MPa for S3, and 7.9 MPa for S4. Lower contact angles indicate higher hydrophilicity of the scaffold. Novel membrane formulations, such as chitosan-alginate-dicalcium phosphate-coated poly(lactic acid), have been developed to control biological conditions and promote cell growth. Furthermore, the creation of sodium alginate/chitosan nanocomposite scaffolds incorporating zircon nanoparticles, hydroxyapatite, and alendronic acid illustrates advancements in scaffold design for bone tissue engineering [[38], [39], [40]]. The calcium orthophosphate-containing composites have been investigated for their formulations, properties, and diverse biomedical applications, underscoring the ongoing innovations in material science for regenerative medicine. Mahheidari et al. [36] demonstrate the biological effects of a composite wound dressing comprising alginate, carboxymethyl cellulose, chorion membrane, diopside nanoparticles, and Botox A. This innovative approach aims to leverage the synergistic effects of these components to accelerate wound healing and improve tissue regeneration. The inclusion of Botox A, known for its ability to modulate inflammation and promote healing, further underscores the multifaceted strategy employed in this research. Li et al. [41] demonstrated the potential for mass-producing porous nanofiber membranes using PVA combined with sodium alginate and graphene, highlighting the advantages of advanced electrospinning techniques. These membranes exhibit enhanced mechanical properties and can be tailored for various applications, including wound healing. Luo et al. [42] contributed to the field by developing hierarchical mesoporous bioactive glass/alginate composite scaffolds for bone tissue engineering. Their research shows the importance of structural design in enhancing the biological performance of scaffolds, which can inform future developments in wound care materials. The results of this study demonstrate that the addition of diopside nanoparticles to the PVA/alginate scaffold enhanced the mechanical and physical properties of the material, as predicted. The improvements in tensile strength and elastic modulus can be attributed to the reinforcing effect of the diopside particles within the polymer matrix, as reported in similar studies on the incorporation of bioactive ceramics into polymer scaffolds.

**Table 4:** Ionometric results of SBF solution after 28 days of immersion by ICP.

Ion concentration (ppm)	S1 (mg/L)	S2 (mg/L)	S3 (mg/L)	S4 (mg/L)
Si	0.69	0.82	0.85	0.91
Ca	92.55	95.65	95.67	98.15
P	39.58	41.25	41.58	42.64

**Table 5:** Statistical results of mechanical properties.

SAMPLE	Elastic Modulus (MPa)±2	Porosity (%) ±2	Elongation (%)± 0.03	Tensile strength (MPa) ±0.05
S1	3.5	68	0.06	0.37
S2	5.6	66	0.04	0.62
S3	6.8	62	0.035	0.73
S4	7.9	58	0.033	0.78

## 4 Conclusion

Based on the comprehensive evaluation of mechanical properties, surface characteristics, and the density of surface accretions on the sample surfaces, the composite scaffold containing 4 wt% diopside nanoparticles can be considered the optimal choice. This composition enhances the spinning process and facilitates the formation of fibers with a reduced diameter. The results of this study indicate that the sample with 4 wt% diopside nanoparticles significantly improves the elastic modulus from 3.5 MPa to 7.9 MPa and increases the tensile strength from 0.37 MPa to 0.78 MPa. The PVA used in this research has a molecular weight of 26,300 and a density of 1.6 g/cm^3^ at 25 °C. The degree of hydrolysis and polymerization of PVA influences its solubility in water, with PVA having undergone high hydrolysis showing lower solubility. The presence of residual acetate moieties diminishes the intermolecular and intramolecular hydrogen bonding interactions that stabilize the hydroxyl (OH-) groups. Furthermore, the elongation percentage declined from 0.06 to 0.033, while the porosity decreased from 68 % to 58 %. This reduction in porosity aligns with the observed agglomeration and the increase in tensile strength. The weight change analysis reveals that samples with a higher concentration of diopside nanoparticles experience the least weight loss and do not exhibit significant changes in gloss. Furthermore, the wettability of the samples decreased from 151° to 121°.

## Availability of data and materials

The datasets supporting the conclusions of this study are included within the article.

## Declaration of competing interest

The authors declare that they have no known competing financial interests or personal relationships that could have appeared to influence the work reported in this paper.
